# The Implications of Cancer Stem Cells for Cancer Therapy

**DOI:** 10.3390/ijms131216636

**Published:** 2012-12-05

**Authors:** Wenjing Jiang, Jianhua Peng, Yue Zhang, William C. S. Cho, Kunlin Jin

**Affiliations:** 1 Department of Otolaryngology, First Affiliated Hospital, Wenzhou Medical College, Wenzhou 325000, China; E-Mails: jiangwen19870810@163.com (W.J.); pjh7218@gmail.com (J.P.); 2 Department of Clinical Oncology, Queen Elizabeth Hospital, Kowloon, Hong Kong; E-Mail: williamcscho@gmail.com; 3 Department of Pharmacology and Neuroscience, Institute for Aging and Alzheimer’s Disease Research, University of North Texas Health Science Center, Fort Worth, TX 76107, USA

**Keywords:** cancer stem cells, differentiation, progenitor, signaling pathway, tumorigenesis

## Abstract

Surgery, radiotherapy and chemotherapy are universally recognized as the most effective anti-cancer therapies. Despite significant advances directed towards elucidating molecular mechanisms and developing clinical trials, cancer still remains a major public health issue. Recent studies have showed that cancer stem cells (CSCs), a small subpopulation of tumor cells, can generate bulk populations of nontumorigenic cancer cell progeny through the self-renewal and differentiation processes. As CSCs are proposed to persist in tumors as a distinct population and cause relapse and metastasis by giving rise to new tumors, development of CSC-targeted therapeutic strategies holds new hope for improving survival and quality of life in patients with cancer. Therapeutic innovations will emerge from a better understanding of the biology and environment of CSCs, which, however, are largely unexplored. This review summarizes the characteristics, evidences and development of CSCs, as well as implications and challenges for cancer treatment.

## 1. Stem Cells and Cancer Stem Cells (CSCs)

Stem cells, which are rare in most tissues, are defined as cells with the ability to perpetuate themselves by self-renewing and to differentiate into a variety of specialized cells in a tissue or organ [1,2]. Both self-renewal and differentiation potential are intrinsic properties of stem cells. The self-renewal of stem cells is involved in duplex implications. One the one hand, stem cells can proliferate infinitely and maintain autologous characteristics. In addition, these stem cells can also be at a quiescent state. However, not all pluripotent stem cells have the self-renewal potential equivalently, indicating that the developmental potential of stem cells will be progressively restricted through the stepwise differentiation along their maturation pathways to generate a hierarchy of progenitors or precursors, which has been verified in the hematopoietic stem cells [3]. On the other hand, stem cells have clone potential, suggesting that stem cells can generate cells phenotypically similar to themselves through genetic replication. Differentiation potential of stem cells is the ability to differentiate into various cell types. The proliferation rate of stem cells is usually slow. Two split pathways, termed symmetry and asymmetry division, are implicated in controlling the probability of self-renewing *versus* differentiation divisions. The former can be divided into two progeny stem cells or differentiated progenitors and thus controlling the self-amplification of stem cells, whereas the latter produces one differentiated progenitor and another daughter [4]. Generally, stem cells can be divided into embryonic stem cells (ESCs) and adult stem cells (ASCs) according to their primary origins. Totipotent ESCs can differentiate into all the tissues. ASCs, also termed tissue stem cells, are a class of pluripotent cells that exist in a variety of organs. Additionally, ASCs take responsibility for the regeneration and repair of tissues [1]. Stem cells exist in a particular environment *in vivo*, and the microenvironment plays an important role in the function preservation of stem cells [5].

CSCs are a small subpopulation of tumor cells with an infinitely proliferative potential existing in tumor tissues [1]. They play a vital role in the early stage of tumor formation and growth, while other tumor cells with restricted or no proliferative capacity will die eventually after a brief split. Consistent with this notion, Clevers [6] proposed that a bulk of tumor consisted of rapidly proliferating, postmitotic and differentiated cells, and that however, only the first-class cells had the self-renewal potential, which are termed as CSCs. The number of CSCs appears to be very low in most tumors, except that it may comprise up to 25% of the total mass in melanoma [7]. Recent studies show that CSCs are the progenitor and real “seed” of tumor cells, representing the main biological characteristics in some tumors [8]. Another viewpoint involved in CSCs concept postulated that the growth of tumors was fueled by a limited amount of cells that were capable of self-renewal [6]. CSCs were also correlated with some specific cell markers, and most of them were similar to markers that were significantly involved in generation, development, oncogenicity, metastasis and recurrence of malignant tumors [9]. Yet, the role of CSCs in multistage cancer progression, particularly with respect to metastasis, has not been well-defined [10]. Recent studies indicated that small subpopulations of tumorigenic pancreatic cancer [11] and colon cancer [12] cells were enriched for the capacity to metastasize. Some CSCs have the ability to form and maintain tumors. It has been proven that CSCs can develop new metastases several years after curative treatment of a primary tumor, which may be explained by the drift of CSCs. For instance, metastatic relapse in breast cancer can occur more than a decade after initial treatment [13]. Owing to more genetic instability, CSCs are easier to adapt to the new environment [14]. Despite the role of CSCs in forming tumor cells with different differentiation ability and maintain tumor uninterrupted growth, the proliferation and differentiation of CSCs are in disorder and out of control [15,16].

CSCs and stem cells have a host of similar characteristics, such as self-renewal, indefinite self-replication, asymmetric cell division, generating a large number of differentiated cells and expressing specific molecules [17,18]. Additionally, stem cells and CSCs have lots of extremely similar regulatory factors that modulate self-renewal, differentiation and the process of proliferation [19]. However, both of them are able to slumber for prolonged periods of time. The difference between CSCs and stem cells is that stem cells function under control, whereas the division and differentiation in CSCs are out of control, which ultimately generate a large number of tumor cells to maintain the tumor growth and heterogeneity via continuous self-renewal and differentiation [20,21].

## 2. Evidence of CSCs

Growing evidence has shown that tumors are derived from and maintained by a rare population of dysregulated stem cells. The CSC hypothesis was first raised by Mackillop *et al.*[22] in 1983. He proposed that there might be a small cluster of cells with similarly special functions to stem-like cells in all the tumors. The first conclusive evidence for CSCs was published in 1997 by Bonnet and Dick. They isolated a subpopulation of leukemic cells that express a specific surface marker CD34, but lack the CD38 marker (CD34^+^/CD38^−^) [23]. After transplantation into mice with severe combined immune deficiency (SCID), these CD34^+^/CD38^−^ cells can form tumors that phenotypically resemble the patient’s original tumor [23,24], indicating that they are tumorigenic. At present, this method has become the gold appraisal standard for identification of CSCs [25]. This notion has subsequently been verified in several solid tumors, including cancers of the head and neck [26], lung [27,28], liver [29], ovary [30], colon [31], pancreas [32] (Table 1). All of these evidences demonstrate that there may be CSCs existing in the tumor tissues, which perform as the driver in the survival process of tumors.

Further evidence of CSCs comes from histology and immunocytochemistry studies. For example, many tumors are very heterogeneous and contain multiple cell types native to the host organ. Heterogeneity is commonly retained by tumor metastases, which implies that the cell that produced them had the capacity to generate multiple cell types. Ginestier *et al.*[42] showed that aldehyde dehydrogenase (ALDH)-positive cells isolated from human breast tumors contained CSCs, as these cells could generate tumors in NOD/SCID mice. Subsequently, Douville *et al.*[43] confirmed that ALDH1 activity can be used to identify and isolate CSCs of the mammary gland and breast cancer. In addition, ALDH-positive CSCs from the colon [33], brain [44], and liver [45] were also capable of forming tumors in immuno-compromised NOD/SCID mice, whereas ALDH-negative cells did not. OCT4 and SOX2, a class of nuclear proteins [46], are both crucial markers to maintain the pluripotent state of stem cells. In addition, both of them and some other factors are expressed in pluripotent stem cells [47,48]. In 2010, we found that ESC protein markers CD133^+^, SOX2 and OCT4 were expressed in a small subpopulation of cells in human primary nasopharyngeal carcinoma (NPC) [49]. Further study showed that these cells were proliferative.

According to label-retaining cell (LRC) trial, adult stem cells can be identified based on their ability to retain nucleoside analog, such as bromodeoxyuridine. In accordance with this principle, Zhang *et al.*[50] found that a few of LRCs existed in human NPC tissues, such as the nasopharyngeal mucosal basal parts and the NPC cell lines. These cells can further develop into tumors after transplantation into the notum of nude mice. In a recent study, laser capture microdissection is used to isolate pure cell populations from NPC and normal nasopharyngeal epithelial tissue samples. Cheng *et al.*[51] confirmed that stathmin, 14-3-3ó, and annexin I are related to differentiation degree and/or metastatic potential of the NPC cell lines.

## 3. CSCs and Cancer Progenitor Cells

Growing evidences suggests the existence of a dynamic equilibrium and bidirectional conversion between CSCs and cancer progenitors [52]. On the one hand, CSCs could self-renew and generated more differentiated cancer progenitor cells hierarchically through asymmetric replication. On the other hand, cancer progenitor cells had the capacity to dedifferentiate and acquire a stem-like phenotype by a series of mechanisms, such as the microenvironment, signaling pathways, molecular circuitries and epigenetic modifications. This could be found in chronic myelogenous leukemia, which showed that a lineage-restricted progenitor or mature cell can acquire stem cell privileges after oncogenic transformation [53–55]. However, what is essential for the events has not yet been determined. Proia *et al.*[56] demonstrated that progenitor cell fate and tumor phenotype could be significantly impacted by the genetic background of patient populations and incidence rates. Understanding the linkages between CSCs and cancer progenitor cells is critical for the development of therapeutic strategies for tumors by inactivating the endogenous dedifferentiation mechanisms.

Cancer progenitor cells display low a self-renewal capacity and a higher probability of terminal differentiation compared with CSCs [52]. Various studies indicated that the majority of leukemic cells descend from a relatively small pool of progenitor cells with high proliferative activity [57]. Additionally, leukemic clone may be organized to generate large numbers of “differentiated” non-proliferative leukemic cells [58]. Take acute myeloid leukemia (AML) colony-forming units for example, these progenitor cells have mainly two properties as listed below: (1) Actively cycling and proliferating *in vivo*, whereas most daughter cells exited from the cell cycle are not able to proliferate *in vitro*; (2) Differentiation to a limited extent *in vitro*, and the capacity can be analyzed by special surface markers at the different differentiation stages. During this process, the cellular morphology was often bizarre and maturation is incomplete. Another study [59] showed that leukemic blast populations, considered as the earliest progenitors, progressively reduced the proliferation and renewal capacity. However, these changes were not associated with morphological evidence of specialization.

Growing evidences indicated that drug-targeted therapies to control tumors either at CSCs or cancer progenitor cells level exhibited different sensitivity. Chronic myeloid leukemia (CML) stem cells were insensitive to tyrosine kinase inhibitors like imatinib, dasatinib and nilotinib, while sensitive to leukemia progenitor cells [60–62]. The mechanism of escaping imatinib inhibition in CML leukemic stem cells might be mediated through the activation of survival pathways such as Wnt/β-catenin and AKT/PTEN pathways [63]. Dasatinib targeted an earlier progenitor population than imatinib in primary CML but did not eliminate the quiescent fraction [61]. Primitive, quiescent Ph^+^ stem cells from CML patients were insensitive to STI571 *in vitro*, thereby these immature Ph^+^ progenitor cells can survive, while the overall sensitivity of CML CD34^+^ progenitor cells to STI571 is mainly determined by cell cycle status [60].

Increasing evidences showed that the surface protein markers expressed by CSCs and cancer progenitor cells were somewhat dissimilar. In 2003 [40], CD44^high/+^ CD24^low/−^ expression was found in breast tumor-initiating stem-like cells. However, it was not clear whether CD44 and CD24 consistently distinguished tumorigenic from non-tumorigenic cells. Subsequently, these CSC-like cells were verified intrinsically resistant to conventional chemotherapy [64] and ionizing radiation [65]. Jiang *et al.*[66] suggested that BCR-ABL transcript levels may be up to 200-fold higher in the most primitive CML progenitors, compared to more differentiated cells. In addition, a study by Venugopal *et al.*[67] showed that brain tumor initiating cells might generate all neural cell types through differentiation. During this period, CD133^+^ stem and early progenitor cells lost their CD133 expression, giving rise to late progenitors and finally differentiated progeny. These lineage programs for cell fate determination can be restricted by PcG proteins, such as Bmi1, which regulates tumor initiation in CD133^+^ stem and early progenitor cells, while regulates tumor maintenance of proliferation, differentiation and cell fate determination in CD133^−^ proliferative progenitors. Likewise, Stewart *et al.*[68] found that CD133 expression changed in ovarian cancer cells during passaging, suggesting that CD133 only marked ovarian CSCs under defined conditions and the hierarchical organization in ovarian cancers was not stable.

## 4. Origins of CSCs

To date, the cell of origin of CSCs remains to be a pendent and troubled problem around the world. There are two hypotheses for the origin of CSCs [69]. One states that CSCs come from normal adult stem cells through an initial genetic mutation, another states that CSCs originate from already differentiated primary cells or differentiated cells that dedifferentiate. Stem cells existed in normal adult tissues may be the targets of carcinogenesis and tumor transformation. Although the number of stem cells is very small, they can progress continual division for a long time and are more likely to accumulate the molecular mutations that cause tumorigenesis. Thus, they are in a tendency of high deterioration. As mentioned above, the phenotype of tumor initiating cells, CD34^+^/CD38^−^ cells, in leukemia is similar to normal hematopoietic progenitor cells [23]. The evidence from hematopoietic system indicates that the genetic mutations in progenitor cells can reactivate self-renewal, suggesting that CSCs may come from other origins, although normal stem cells are found in many solid tumors.

### 4.1. From Stem Cells

It has been proposed that CSCs and normal stem cells can interconvert into each other. The more important consequence of this event is that normal stem cells can generate CSCs that ultimately induce a new tumor. Emerging evidence has supported this notion, as CSCs share many properties of normal stem cells. For examples, both have the capacity of self-renewal and non-directional differentiation potential, and many classic cancer related signal transduction pathways also regulate the development of normal stem cells. In this scenario, cancer cells could simply utilize the existing stem cell regulatory pathways to stimulate their self-renewal. In addition, both stem cells and CSCs have telomerase activity and amplified telomere repeats, while most adult human somatic cells lack detectable telomerase. Another theory associates stem cells with the formation of tumors, which is most often related with tissues with a high rate of cell turnover. In these tissues, it has long been expected that stem cells are responsible for tumor formation. Tissue with fast renewal, such as epithelial tissue and those of the hematopoietic system, are sites with high incidence of cancer. The faster tissues renew, the higher the rate of mutation that will occur during replication and transcription. Although it is not clear which target cells mutate and transform to tumors, experimental data obtained from a variety of tumors show that certain colon cancers and leukemia result from an accumulation of multiple mutations of stem cells [70]. Due to the heterogeneous nature of evidence, it is possible that any individual cancer could come from an alternative origin. Another hypothesis is that the developing stem cells are mutated and then expand such that the mutation is shared by many of the descendants of the mutated stem cell. These daughter stem cells are then much closer to becoming tumors, and many of them have more chance of a mutation that can cause cancer [71]. Taken together, these findings suggest that there may be some linkages between CSCs and stem cells.

### 4.2. From Progenitor Cells

Some researchers presume that CSCs may be obtained by the mutation of committed progenitor cells with an ability of self-renewal. For example, leukemia stem cells can be transformed from granulocyte-macrophage progenitors with the assistance of MLL-AF9 fusion protein [72]. Another study also shows that neuronal progenitor cells are likely to be the target of carcinogenic mutations [73]. All of these results indicate that the CSCs may originate from the committed progenitor cells.

### 4.3. Other Possible Sources

Despite the lack of direct experimental evidence, some studies show that CSCs may be the fusion of stem cells and other cells [74]. These new integration cells obtain the capacity of self-renewal, and are thus effortless to accumulate more mutations for canceration. For example, bone marrow derived cells can fuse with epithelial tissue tumors [75]. Additionally, a recent study [76] concerned with migrating CSCs showed that the development of tumor metastasis might correlate with the dissemination of CSCs, which were mainly caused by the cells at the tumor margins that have undergone epithelial-mesenchymal transition (EMT). The linkages of EMT and the emergence of stem cells have also been reported by Mani *et al.*[77]. The CSC hypothesis presume that the path via which CSCs self-renew and generate more differentiated neoplastic progenitor cells through asymmetric replication is hierarchical and unidirectional. However, emerging evidences are beginning to support the notion that relatively differentiated progenitors could switch to dedifferentiate and acquire a stem-like phenotype in response to either genetic manipulation or environmental cues [52], which has been identified sequentially in mammary carcinoma cells [77], A549 lung cells [78], colon cancer cells [79] and glioblastoma cells [80]. Terminally differentiated cells including human somatic cells and skin cancer cells can be artificially induced through specific transcriptional networks to reprogram pluripotent ESCs, called induced pluripotent stem cells (iPSCs), which is a significant breakthrough against the dogma that differentiated cells is irreversible [81–83]. However, these iPSCs are tumorigenic, suggesting that oncogenic transformation of partially differentiated cells can lead to the emergence of CSCs. In addition, more recent studies show several plausible origins of CSCs. For examples, (1) lineage tracing reveals that lgr5^+^ cells could generate additional lgr5^+^ cells as well as all other adenoma cell types, thus exhibiting activity of CSCs in mouse intestinal adenomas [84]; (2) the restricted subpopulation, with properties similar to those proposed for CSCs, propagates glioblastoma growth after chemotherapy [85]; (3) using an inducible genetic lineage tracing system, Gregory Driessens *et al.* found that the yellow fluorescent protein (YFP) could been expressed in around 1% of basal papilloma epithelial cells in mice, and these YFP-labeled tumor cells were capable of generating all cell types that comprised the tumor [86].

## 5. Molecular Mechanisms Controlling CSCs

At present, the molecular mechanisms underlying regulating the development of CSCs remain to be unexplored. Various signaling pathways have been suggested, and some of them are reviewed as follows.

### 5.1. Notch Signaling Pathway

The Notch signaling pathway is a highly conserved cell signaling system present in most multicellular organisms, which regulates widely the development and homeostasis of vertebrate and invertebrate embryos and adult individuals through the local interaction between cells, and controls how cells respond to intrinsic or extrinsic developmental cues that are necessary to unfold specific developmental programs [18]. Notch activity affects the implementation of differentiation, proliferation, and apoptotic programs, providing a general developmental tool to influence organ formation and morphogenesis [87].

Studies indicate that Notch signaling is likely to be implicated in the pathogenesis of many human tumors, including leukemia [88] and pancreatic cancer [89]. Additionally, accumulated evidence demonstrated that Notch signaling might contribute to cancer metastasis [90]. More significantly, the Notch pathway plays a critical role in the linkages between angiogenesis and CSCs self-renewal and is thus receiving increased attention as a target to eliminate CSCs [91]. The self-replication and tumor formation capacity of leukemic CSCs is reduce by blocking Notch signaling activation, and conversely, it will promote growth and differentiation of glioma CSCs [92]. A recent study shows that γ-secretase inhibitors can render the glioma CSCs more sensitive to radiation at clinically relevant doses; thereby inhibition of Notch signaling holds promises to improve the efficiency of current radiotherapy in glioma treatment [93–95]. In 2010, we found that Notch1-activated form and its downstream target were expressed in SOX2- and OCT4-positive cells in human NPC [49], suggesting that Notch1 signaling was activated in these cells and might involve in molecular regulation of cancer stem/progenitor-like cells in NPC. Therefore, targeting Notch signal transduction pathway may bring us an innovative therapeutic strategy for cancer treatment by eliminating cancer stem/progenitor cells.

### 5.2. Wnt/β-Catenin Signaling Pathway

Wnt proteins are secreted signaling molecules of Wnt signaling, and nuclear β-catenin function as a key mediator. One indicator of Wnt pathway activation is the nuclear accumulation of its main effector β-catenin, which is one component of a transcriptional activation complex that includes members of the T-cell factor/lymphoid enhancer factor (TCF/LEF) family of DNA binding proteins [96]. In normal cells the transcriptional regulator β-catenin is tightly controlled by a multiprotein complex that contains the tumor suppressor adenomatous polyposis coli (APC) [97]. Activation of Frizzled receptors by Wnt ligands could disrupt this complex and thus results in the translocation of β-catenin to the nucleus, where it associates with the TCF/LEF family of transcription factors [98,99]. APC mutations generally result in a defective β-catenin degradation complex and β-catenin accumulation in the nucleus [100].

Wnt signaling pathway regulates many developmental processes through transcriptional regulation [101] and its dysregulation is a key factor for the initiation of various tumors [102]. Additionally, there are broadly increasing evidences that Wnt/β-catenin signaling is involved in the molecular mechanism underlying controlling CSCs. Studies document that Wnt signaling is activated in response to DNA damage [103] and genomic instability may drive the malignant transformation of nontumorigenic stem cells to glioblastoma CSCs [104]. Survivin, a transcriptional target of β-catenin, promotes cellular survival in response to apoptotic stimuli [105]. Increased survivin expression by β-catenin can impose a stem-cell phenotype in colorectal cancer cells [105,106]. In the absence of pathway stimulation, β-catenin protein is destabilized by a cytoplasmic complex containing the proteins Axin, APC and glycogen synthase kinase-3b [107]. The reduced β-catenin levels permit repression of Wnt target genes by association of transcriptional co-repressors with TCF/LEF [107,108]. In 2007, Zhao *et al.*[109] demonstrated that deletion of β-catenin might lead to a reduced ability of BCR-ABL, therefore impairing the renewal of normal and CML CSCs in mice. In 2010, Vermeulen *et al.*[110] documented that Wnt signaling activation was a marker for colon CSCs and was regulated by the microenvironment. Meanwhile, they also found that myofibroblasts play an important role in installing and maintaining colon CSC fate through the regulation of Wnt signaling, indicating that these factors could enhance Wnt signaling and reinstall features of stemness in more differentiated tumor cells. Additionally, Wnt and β-catenin signaling may contribute to radioresistance of CSCs [111,112].

### 5.3. Other Signaling Pathways Implicated in CSCs

Other molecular pathways or factors that play a critical role in the development of CSCs include following: (1) mTOR signaling pathway, which is frequently aberrantly activated in human cancers, and significantly correlated with biological cell behaviors [113]. Recent studies show that mTOR signaling may be involved in mechanisms underlying the regulation of biological behaviors of cancer stem-like cells. The mTOR pathway is explicitly correlated with the survival and the proliferation of cancer stem-like cells in human breast cancer by specific pathway inhibitors, gene knockdown and tumorigenicity assays *in vivo*[114]. In addition, the reinforcement of mTOR signaling in medulloblastoma CSCs may contribute to radioresistance of these cells, and contrarily, mTOR inhibition could increase radiosensitivity [115]; (2) Fibroblast growth factors (FGFs), which comprise a large family of signaling molecules with various functions in development as well as in adult [116]. In addition, these factors are also useful for culturing CSCs derived from various types of human tumor tissues, such as brain [117] and gastric tumors [118]. Studies indicate that FGF-2 accumulation and activity are important for the maintenance of the undifferentiated phenotype of leukemic stem/progenitor cells [116]. Using the CSC model, the dysregulation of the FGF-2 pathway in malignant cells may provide growth advantage and self-renewal stimulation to CSCs; (3) Sonic hedgehog (SHH) signaling, which is one of the key regulators of animal development. A recent study demonstrates that SHH signaling regulates the expression of stemness genes and the self-renewal of CD133^+^ glioma CSCs [119]. In addition, CSCs in human gliomas also require SHH pathway activity for their proliferation, survival and tumorigenicity [107]; (4) Recent studies [120] suggest that there is an association between the expression of the stem cell marker ALDH1 and HER2 amplification in breast tumors, and the clinical efficacy of HER2-targeting agents may correlate with their ability to target breast CSCs. The addition of the HER2-targeting agent lapatinib to chemotherapy reduces the CSC number. Contrarily, transfection of HER2 into breast cancer cell lines increases the CSC population and results in increased invasion and metastasis. Strikingly, one third of HER2-positive tumors do not respond to HER2-targeting agents, which could be attributed to aberrant activation of the downstream PI3K/Akt pathway. This suggested that inhibiting Akt downstream of HER2 signaling may effectively target breast CSCs in HER2-resistant tumors [121]; (5) Epidermal growth factor, which is a key growth factor used in culturing and maintaining cancer stem cells [122]. This notion makes it possible to treat chemotherapy-resistant breast CSCs with the epidermal growth factor receptor inhibitor lapatinib [64]; (6) Bao *et al.*[123] in 2008 had identified that L1CAM as a differentially expressed surface glycoprotein was expressed and linked to therapeutic resistance in glioblastoma stem cells (GSCs). Targeting L1CAM with shRNAs specifically disrupted tumor-sphere formation and growth of GSCs *in vitro* (7). The signal transducer and activator of transcription 3 (STAT3) is a crucial transcriptional regulator involved in tumorigenesis. Inhibition of STAT3 with specific inhibitors or targeting STAT3 with specific shRNAs disrupts proliferation and maintenance of GSCs [124,125].

## 6. Implications for Cancer Treatment

Once a cancer has been diagnosed, treatments vary according to cancer type and severity. Surgery, radiation therapy, chemotherapy or hormonal therapy represents traditional approaches designed to remove or kill rapidly-dividing cancer cells. However, there has been hardly any substantial progress with new therapies regarding clinical endpoints, despite significant advances in molecular mechanisms of cancer. Cancer remains a major public health issue. Conventional anti-cancer treatments target the more mature cancer cells that form the bulk of the tumor, but do not target the CSCs, which are relatively quiescent and intrinsically resistant, thus possibly accounting for treatment failures [126]. To target tumors effectively with minimal toxicity, drugs that specifically target the relatively rare CSC subpopulation need to be identified [127].

Tumor metastasis is a complex process, and is also the main cause of the death of cancer patients in clinic. It is the key to improve the prognosis of patients by removing CSCs selectively with no significant toxicity [128]. Several pieces of instances have been expounded surrounding this conclusion: (1) The maintenance of CSCs viability can be influenced by the microenvironment, thereby appropriate microenvironment exhibits vital importance for CSCs, which brings us a new insight into oncotherapy by changing the survival microenvironment. For example, glioma CSCs have been found congregated close to capillaries in a niche, thus, vasculature-targeted therapeutic strategies could effectively destroy the niche and eradicate the tumor [129]; (2) Growing evidences indicate that CSCs regulate some pathways of normal stem cell self-renewal and the continuing expansion of self-renewal could consult in tumorigenesis. Accordingly, the exploration of self-renewal pathways about defective cancer cells may provide us a new treatment for cancer; (3) Potential approaches to killing CSCs also include inducing tumor cell differentiation in addition to blocking self-renewal signaling and inhibiting cell survival mechanisms. For example, renal CSCs can be differentiated into epithelial cells after treatment with interleukin-15. The differentiated epithelial cells derived from renal CSCs are sensitive to chemo-therapeutic drugs [130]. Knockdown of CD44 caused BCSCs to differentiate into non-breast CSCs with lower tumorigenic potential, and altered the cell cycle and expression profiles of some stem cell-related genes, making them more similar to those seen in non-breast CSCs and resulting in a loss of stemness and an increase in susceptibility to chemotherapy or radiation [131]. As described above, some of the signaling pathways for the differentiation of normal stem cells may be maintained in cancer stem cells. Wnt signaling plays an important role in maintaining the pluripotency of human ESCs and is also implicated in sustaining CSC phenotype by dedifferentiating mechanisms [132]. In 2007, Wei *et al.*[133] confirm that the Wnt pathway plays a critical role in the self-renewal and maintenance of stem cells. A recent report documents that rapamycin-mediated inhibition of mTOR signaling may prevent CSC self-renewal and circumvent CSC-mediated resistance to cancer therapeutics [134]; (3) Some studies show that patients with tumors that express high levels of molecules associated with CSCs had a poorer prognosis than patients with tumors that express low levels of these markers [135]. In breast cancer, for example, the most poorly differentiated tumors have the highest burden of CSCs [136]. Subsequent study indicated that metformin not only selectively killed existing CSCs, but also indirectly lowered the CSC burden by inhibiting the conversion of non-stem cancer cells to CSCs [137]. Cell differentiation is regulated, at least in part, by a recent discovered class of molecules-microRNAs (miRNAs), and as a consequence, a potential therapeutic use of miRNAs is to correct these aberrant transcript levels involved in the signaling pathways of cancer cells [138], especially CSCs [139,140]; (4) To overcome the chemotherapy resistance of CSCs through the activity of multiple drug resistance (MDR) transporters. Recent study indicates that salinomycin, a specific inhibitor of P-glycoprotein, can restore a normal drug sensitivity of MDR cell lines and induce CSC death [141].

**Figure 1:**
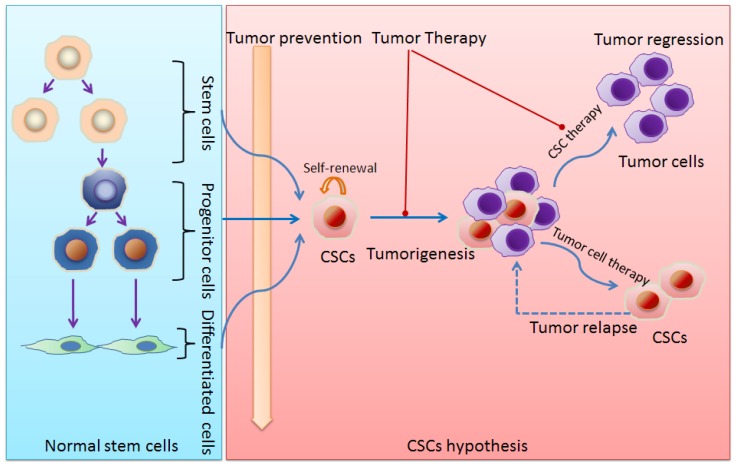
Schematic diagram of stem cells and cancer stem cells (CSCs). **Left panel**: Normal stem cell proliferation and differentiation. **Right panel**: CSCs and tumorigenesis as well as implications of CSCs for cancer therapy.

Another way to control the tumor progression is to induce differentiation of CSCs. Study by Piccirillo *et al.*[142] showed a reduction of the number of glioma CSCs after treatment with bone morphogenetic proteins. Additionally, the quiescent CSCs were involved in the resistance of CSCs to anti-cancer treatments as discussed above. Therefore, it will be of great importance to explore the means that break the quiescent state of CSCs. Studies by Ishikawa and his colleagues have recently induced AML stem cell cycle entry and increased the sensitivity of these cells by using granulocyte colony-stimulating factor treatment [143]. The CSC concept promises the development of therapeutic strategies beyond traditional anti-proliferative agents (Figure 1). Studies have confirmed that potential approaches to kill CSCs may exploit the survival mechanisms of the CSCs [6]. The biological exploration that correlative with CSCs in solid tumors will bring us new viewpoints to the clinical diagnosis, treatment, CSC-targeted drug researches and the preclinical trials. Moreover, it will be better to predict the results of clinical treatment by assess the behavior of CSCs. At present, although the cancer treatments which target CSCs unveil a new prelude, it is still a problem that how to identify the CSCs, especially to prevent its formation. The therapeutic significances of CSCs against solid tumors are summarized in Table 2.

## 7. Conclusions

Recent studies have found that CSCs are the main reason for tumor growth, recurrence and metastasis [144]. Dingli and Michor [145] suggests that successful therapy must eradicate CSCs. To achieve the maximum effect and eradicate a tumor, the CSCs compartment should be targeted specifically. However, some properties of CSCs make them difficult cells to kill, as listed below: (1) To date, although many CSCs markers have been found, it is still impossible to take them as candidates for antibody therapy owing to their broad expression in healthy tissue. Additionally, transient and long-term dormancy are generally believed to be fundamental characteristics of CSCs [146,147], and the latter may be crucially involved in the resistance of CSCs to anti-proliferative chemotherapy. To probe dormancy of breast CSCs, Pece *et al.*[136] in 2010 searched a gene signature for cultured quiescent mammary gland stem cells according to their ability to retain the lipophilic dye PKH26. Subsequently, this gene signature was verified correlative with CSC behavior after applied to breast cancers. However, it is still unclear how to explore the existence of dormant CSCs exactly by overcoming technical challenges. (2) CSCs maintain the property of anti-tumor therapies through the activity of multiple drug resistance transporters, which will decrease the effective drug concentration within the cells by pumping drugs out of the cells [148]. (3) Many anti-cancer drugs cause direct damage to the structure of DNA, and resistance to these drugs reactively results from activation of DNA repair systems in CSCs [149]. (4) Meanwhile, Wang *et al.*[150] and Liang *et al.*[151] have observed the phenomenon that CSCs affect radiation sensitivity. CSCs are linked to radiation resistance and angiogenesis [101,152,153], which affect the treatment’s effectiveness. CSCs in therapeutic resistance and angiogenesis have better survival skills [145].

Despite recent clinical studies have begun to monitor the behavior of CSCs during chemotherapy, it is still an urgent requirement of more clinical studies to assess how responses to therapy correlate with CSC biomarkers. Development of new CSC-targeted strategies is currently hindered by the lack of reliable markers for the identification of CSCs and the poor understanding of their behavior and fate. Although cancers represent a major therapeutic challenge, with better understanding in the CSCs, the more specific markers to look for this lethal disease. There is no doubt that the application of CSC theory to study the tumorigenesis mechanisms will lead a paradigm shift in the cancer research and the understanding of the essence of cancer, supplying a new way to effectively diagnose tumor sites and find functional proteins as potential therapy targets.

## Figures and Tables

**Table 1:** Cancer stem cell (CSC) markers identified in several solid tumors.

Cancer Types	Cell Surface Markers	Reference
Lung cancer	CD24^+^, CD44^+^, CD133^+^	[27,28]
Hepatic carcinoma	CD90^+^, CD45^−^, (CD44^+^)	[29]
Ovarian cancer	CD44^+^, CD117^+^	[30]
Colon cancer	CD133^+^, EpCAM^+^, CD44^+^, CD166^+^, ALDH1^+^	[31,33–35]
Pancreatic cancer	CD44^+^, CD24^+^, ESA^+^, CD133^+^	[32]
Melanoma	ABCB5^+^	[36]
Ewing’s sarcoma	CD133^+^	[37]
Glioma	CD133^+^	[38]
Sarcomas	CD105^+^, CD44^+^, Stro1^+^	[39]
Breast cancer	CD44^+^CD24^−/low^	[40]
Prostate cancer	Sca1^+^, CD133^+^, CD44^+^	[41]
Head & neck squamous cell carcinoma	CD44^+^	[26]

**Table 2:** The therapeutic significance of cancer stem cells (CSCs) against solid tumors.

Achieving the maximum effect and eradicating a tumor Changing the survival microenvironment of CSCs Blocking the activation of pathways Correcting aberrant transcript levels Inducing differentiation and death of CSCs Breaking the quiescent state of CSCs Increasing the sensitivity of CSCs Exploiting the survival mechanisms of the CSCs Giving new viewpoints to the preclinical trials and clinical medicine
